# Prevalence of Illicit Drug Detection in 5 US Cities Among Out-of-Treatment People Who Inject Drugs

**DOI:** 10.1001/jamanetworkopen.2025.55882

**Published:** 2026-02-05

**Authors:** Nabila El-Bassel, Steven Shoptaw, Timothy Skalland, Brett Hanscom, William Clarke, Mark A. Marzinke, Jessica M. Fogel, Paul Richardson, Rahul Paul Choudhury, Cecile Denis, David Goodman-Meza, Irene Kuo, Jordan E. Lake, Ellen A. B. Morrison, Amy M. Richards, Jayla Harris-Wisecarver, Melissa Cummings, Redonna Chandler, Philip Andrew

**Affiliations:** 1 School of Social Work, Columbia University, New York, New York; 2 Department of Family Medicine, University of California, Los Angeles; 3 Vaccine and Infectious Disease Division, Fred Hutchinson Cancer Center, Seattle, Washington; 4 Department of Pathology, Johns Hopkins University School of Medicine, Baltimore, Maryland; 5 Department of Medicine, Johns Hopkins University School of Medicine, Baltimore, Maryland; 6 Statistical Center for HIV/AIDS Research, Fred Hutchinson Cancer Center, Seattle, Washington; 7 Department of Psychiatry, Perelman School of Medicine, University of Pennsylvania, Philadelphia; 8 Kirby Institute, University of New South Wales, Sydney, New South Wales, Australia; 9 Department of Epidemiology, Milken Institute School of Public Health, George Washington University, Washington, DC; 10 UTHealth Houston, Houston, Texas; 11 ICAP, Mailman School of Public Health, Columbia University, New York, New York; 12 FHI 360, Durham, North Carolina; 13 Addiction Policy Forum, Bethesda, Maryland

## Abstract

**Importance:**

In the US, the overdose crisis continues to be driven by fentanyl, xylazine, and stimulant-involved polysubstance use among people who inject drugs, especially those who are not engaged in medical care.

**Objective:**

To estimate the overall prevalence of illicit drugs detected among people who inject drugs in 5 US cities by city and sociodemographic characteristics and assess trends in drug detection over a 2-year period.

**Design, Setting, and Participants:**

This cross-sectional study included data from individuals enrolled in HIV Prevention Trials Network (HPTN) 094, a randomized clinical trial to evaluate an integrated mobile unit engaging adults aged 18 years or older who inject drugs in HIV services between June 2021 and September 2023 in New York City; Houston, Texas; Los Angeles, California; Philadelphia, Pennsylvania; and Washington, DC. All analyses were completed between August 2021 and August 2025.

**Main Outcomes and Measures:**

Baseline prevalence of toxicologic detection was assessed using liquid chromatography–high-resolution mass spectrometry. Differences in toxicologic detection by sociodemographic characteristics (age, race and ethnicity, housing status, and incarceration history), study site, and illicit drug type over time were analyzed using generalized linear models.

**Results:**

Across 444 participants, 303 (68.2%) were male, 267 (60.1%) were aged 30 to 49 years, 203 of 440 (46.1%) were unhoused, and 91 of 442 (20.6%) had a recent incarceration history. In all, 414 participants (93.2%) tested positive for fentanyl, 328 (73.9%) for cocaine, 299 (67.3%) for amphetamine-type stimulants, and 234 (52.7%) for xylazine. Nearly all participants (421 [94.8%]) tested positive for polysubstance drugs (fentanyl or opioids with stimulants, benzodiazepines, cocaine, and/or xylazine). Fentanyl detection was high across all sites; xylazine was most common in New York City (68 of 94 [72.3%]), Philadelphia (111 of 112 [99.1%]), and Washington, DC (31 of 41 [75.6%]). Every 6 months, xylazine detection increased in New York City by 10.3% (95% CI, 4.0%-16.5%; *P* = .001), and its prevalence stayed high in Philadelphia. Amphetamine-type stimulant detection increased in Washington, DC, by 15.0% (95% CI, 2.9%-27.1%) every 6 months over the enrollment period (*P* = .02). Across all sites, cocaine prevalence was higher among unhoused than housed participants (difference, 11.4%; 95% CI, 3.6%-19.2%; *P* = .004), and stimulant detection was elevated among those recently incarcerated vs not (difference, 9.9%; 95% CI, 1.4%-18.5%; *P* = .02).

**Conclusions and Relevance:**

This cross-sectional study found widespread fentanyl and polysubstance detection, with rising xylazine and stimulant detection that varied by sociodemographic and structural vulnerabilities and may be due to adulteration within the unregulated drug supply. These findings highlight urgent public health needs for real-time drug supply surveillance, targeted harm-reduction services, and integrated treatment approaches to reduce overdose risk and address social and structural vulnerabilities.

## Key Points

**Question:**

What is the prevalence of illicit drug detection among people who inject drugs who are not engaged in medical care across 5 US cities?

**Findings:**

In this cross-sectional study of 444 people who inject drugs, fentanyl was detected among 93%, xylazine among 53%, polysubstance drugs among 95%, and amphetamine among 67%; unhoused and recently incarcerated individuals had higher prevalence of cocaine and stimulant detection, respectively. Amphetamine prevalence increased over time in Washington, DC, and Philadelphia had the highest prevalence of xylazine detection.

**Meaning:**

The findings suggest localized, real-time drug surveillance for out-of-treatment individuals who inject drugs, often missed by traditional public health systems, is needed.

## Teaser

This cross-sectional study examines prevalence of and trends in illicit drugs detected over 2 years in 5 US cities among individuals who inject drugs and are not engaged in medical care.

## Introduction

The prevalence of injection drug use in the US remains a significant public health problem. The most recent prevalence estimates (2018) suggest there are 3.7 million people who inject drugs, or 1.5% of the US population, with the greatest burden on males and young populations aged 18 to 29 years.^1^ The overdose crisis disproportionately affects Black or African American adults, particularly those aged 35 to 54 years.^2,3^ Adults aged 55 years or older have experienced the steepest increases in overdose mortality.^2,3^

Incarceration significantly increases overdose vulnerability by disrupting opioid tolerance, limiting access to medications for opioid use disorder (MOUD), and creating a heightened risk of return to drug use upon release.^4^ Overdose death rates peak during the first 2 weeks after release from incarceration among people who use drugs, with overdose risk up to 40-fold greater compared with the general population.^5,6^ Postrelease stressors including housing instability and pervasive drug availability further exacerbate return to drug use.^7,8^ Homelessness is associated with overdose vulnerability, with several studies across the US indicating that homelessness is significantly and positively associated with opioid-involved overdose.^9,10,11^ Research also shows a direct association between the proportion of a population that is unhoused, prevalence of drug use, and overdose mortality, particularly in areas with widespread fentanyl exposure.^10,12^

Extensive research shows that injection drug use is associated with transmission of HIV, sexually transmitted infections, hepatitis C, and fatal and nonfatal overdose.^13,14,15^ Provisional data from the Centers for Disease Control and Prevention (CDC) National Center for Health Statistics indicated a 26.9% decrease in drug overdose deaths across all US regions in 2024 (80 391) compared with 2023 (110 037).^16^ Increased overdose fatality rates among people who use drugs (either injected or noninjected), however, have evolved into the fourth wave of the US drug overdose crisis.^17,18^ The current wave is strongly characterized by polysubstance use, defined as the use of fentanyl, opiates, or synthetic opioids with any amphetamine-type stimulants, benzodiazepines, cocaine, and/or xylazine—an α2 adrenergic agonist and veterinary anesthetic.^17,19^ Most fatalities in the fourth wave are attributed to fentanyl, with or without stimulant use,^19^ and methamphetamines remain a major drug of choice in the western US.^20^ Another important phenomenon of the fourth wave is the transition to smoking or inhaling drugs rather than injecting. Smoking has now surpassed injection as the leading route of ingestion for overdose fatalities.^20,21^

HIV Prevention Trials Network (HPTN) 094 was a randomized clinical trial evaluating an integrated mobile unit to engage people with opioid use disorder who inject drugs and increase use of MOUD and HIV prevention and treatment services in 5 cities (New York City; Houston, Texas; Los Angeles, California; Philadelphia, Pennsylvania; and Washington, DC).^22^ In this study, we examined the toxicologic detection of different types of illicit drugs overall and across the duration of HPTN 094 enrollment. We also examined variations in the prevalence of detected drugs by age, race and ethnicity, sex assignment at birth, geographic location, being unhoused, and history of incarceration. Based on local drug trends,^17,19^ we hypothesized a high prevalence of fentanyl and xylazine detection among participants in the eastern cities (Philadelphia, New York City, and Washington, DC) earlier than participants in the western cities (Houston and Los Angeles).

## Methods

### Study Participants

#### Inclusion and Exclusion Criteria

This cross-sectional study used data from HPTN 094. Participants who met eligibility criteria were invited to participate in a baseline interview and were enrolled between June 2021 and September 2023. All participants completed written informed consent prior to participating in study procedures, and a single institutional review board (Advarra) provided ethical approval for HPTN 094; this cross-sectional analysis was exempt from additional IRB approval. The current study was conducted and reported in accordance with the Strengthening the Reporting of Observational Studies in Epidemiology (STROBE) reporting guideline.^23^

Participants were required to meet the following criteria: (1) be at least 18 years of age, (2) have a urine test positive for recent opioid use and evidence of recent injection drug use (visible venipuncture marks), (3) meet diagnostic criteria for opioid use disorder, (4) be able to give informed consent, (5) be willing to start MOUD treatment, (6) complete an assessment of understanding, (7) have confirmed HIV seropositivity or self-reported sharing of injection equipment and/or condomless sex in the past 3 months with partners living with HIV or with unknown HIV status, and (8) provide locator information. Participants were excluded if they (1) self-reported being prescribed MOUD in the 30 days prior to screening, (2) had a urine test positive for methadone (with the exception of verified hospitalization), or (3) were enrolled in another study.

#### Recruitment Strategies

Prior to study launch, mobile unit study locations were identified using a data-informed, community-centered strategy.^22^ Using local administrative data, neighborhoods were selected based on high overdose rates, significant unhoused populations, and limited access to services for drug use treatment. Surveillance data and a web-based mapping tool allowed sites to analyze local HIV and opioid epidemics and map neighborhood boundaries to determine mobile clinic locations. Field staff visited known hot spots, including locations outside jails; community supervision programs; shelters; syringe exchange sites; detox centers; emergency departments; hospitals; and street-based areas such as tourist areas, parks, and strolling areas.^22^ Although only individuals meeting study eligibility were enrolled, mobile units routinely provided harm-reduction materials, naloxone, and service referrals to all community members regardless of eligibility. These outreach activities were critical to maintaining trust and community engagement.

### Study Assessments

Research staff conducted the baseline interview and collected urine samples. Self-reported sociodemographic characteristics were captured on case report forms. Urine samples collected at enrollment were analyzed at the HPTN Laboratory Center (Johns Hopkins University, Baltimore, Maryland) using liquid chromatography–high resolution mass spectrometry (LC-HRMS) with MS2 library matching (Thermo Fisher Scientific) to assess the presence of methamphetamine or amphetamine, benzodiazepines, buprenorphine, cannabis, cocaine, fentanyl, methadone, opiates, synthetic opioids, and xylazine. Fentanyl was categorized separately from other opiates and synthetic opioids. The “opiate” category included morphine, codeine, and heroin metabolites, while “synthetic opioids” referred to nonfentanyl compounds such as tramadol, U-series analogs, and nitazenes.

Positive results using LC-HRMS testing were defined as any presence of drug detection above the cutoff or limit of detection (more details are provided in the eAppendix in Supplement 1). All results refer to toxicologic detection using LC-HRMS, which indicates exposure rather than self-reported or intentional use.

### Statistical Analysis

Descriptive statistics were used to summarize the prevalence of drugs detected overall, by city, and by sociodemographic characteristic (age, sex at birth, race and ethnicity, current housing status, and incarceration within the past 6 months). Self-reported race and ethnicity categories were Hispanic or Latino, non-Hispanic Black/African American (hereafter, *Black*), non-Hispanic White (hereafter, *White*), and other non-Hispanic race (included American Indian or Alaska Native, Asian, Native Hawaiian or Other Pacific Islander, and multiracial). To compare drug use across demographic groups, binomial generalized linear models (GLMs), adjusted for study site, were used and risk differences were estimated. Participants missing housing status and participants reporting “other” as their race and ethnicity were not included in analyses of drug detection by those factors due to their small sample sizes. Pairwise comparisons were conducted for each drug type and demographic variable, with no multiple-testing adjustments. *P* values are intended to be descriptive and are presented alongside effect sizes (differences in drug-detection rates) and 95% CIs. Two-sided *P* < .05 was considered significant.

To evaluate trends in drugs detected, the enrollment period was subdivided into four 6-month periods (June 2021 to January 2022, February to August 2022, September 2022 to March 2023, and April to September 2023). Binomial GLMs estimating risk differences by city and by drug type were used to assess changes in drugs detected over time. For Philadelphia, the GLMs for fentanyl, xylazine, and polysubstance use over time did not converge due to near 100% prevalence across all enrollment periods for these drugs. In these 3 analyses, normal linear regression models of grouped proportions by enrollment period were used. All analyses were completed between August 2021 and August 2025 using SAS, version 9.0 (SAS Institute Inc).

## Results

### Baseline Sociodemographic Characteristics of Study Participants

Of 447 individuals invited to participate, 3 did not provide a baseline urine specimen, leaving 444 participants for this analysis overall. Across all participants, 141 (31.8%) were female and 303 (68.2%) were male. Of 440 participants with housing data, 203 (46.1%) were unhoused, and 91 of 442 participants with incarceration data (20.6%) had a recent incarceration history (Table 1). Enrollment by city was 94 in New York City (21.2%), 104 in Houston (23.4%), 93 in Los Angeles (20.9%), 112 in Philadelphia (25.2%), and 41 in Washington, DC (9.2%). Most participants were aged 30 to 49 years (267 [60.1%]), ranging from 15 (36.6%) within the Washington DC sample to 83 (74.1%) in the Philadelphia sample. Black individuals (n = 83) made up 18.7% of the participants, with particularly high representation in Washington, DC (33 of 41 participants [80.5%]). Hispanic or Latino individuals comprised one-third of the total enrolled study population (145 [32.7%]), 199 participants (44.8%) were White, and 17 (3.8%) were other non-Hispanic race.

**Table 1.** Baseline Demographics, Overall and by Site

Characteristic	Participants, No. (%)					
	Overall (N = 444)	New York City (n = 94)	Los Angeles (n = 93)	Washington, DC (n = 41)	Houston (n = 104)	Philadelphia (n = 112)
Age group, y						
<30	49 (11.0)	4 (4.3)	9 (9.7)	3 (7.3)	18 (17.3)	15 (13.4)
30-49	267 (60.1)	54 (57.4)	51 (54.8)	15 (36.6)	64 (61.5)	83 (74.1)
≥50	128 (28.8)	36 (38.3)	33 (35.5)	23 (56.1)	22 (21.2)	14 (12.5)
Sex assigned at birth						
Female	141 (31.8)	27 (28.7)	26 (28.0)	9 (22.0)	36 (34.6)	43 (38.4)
Male	303 (68.2)	67 (71.3)	67 (72.0)	32 (78.0)	68 (65.4)	69 (61.6)
Ethnicity and race^a^						
Hispanic or Latino	145 (32.7)	51 (54.3)	51 (54.8)	3 (7.3)	30 (28.8)	10 (8.9)
Non-Hispanic Black or African American	83 (18.7)	24 (25.5)	4 (4.3)	33 (80.5)	8 (7.7)	14 (12.5)
Non-Hispanic White	199 (44.8)	16 (17.0)	35 (37.6)	0	63 (60.6)	85 (75.9)
Other non-Hispanic race^b^	17 (3.8)	3 (3.2)	3 (3.2)	5 (12.2)	3 (2.9)	3 (2.7)
Current housing status, No./total No. (%)						
Housed^c^	237/440 (53.9)	38/94 (40.4)	77/93 (82.8)	29/40 (72.5)	57/101 (56.4)	36/112 (32.1)
Unhoused^d^	203/440 (46.1)	56/94 (59.6)	16/93 (17.2)	11/40 (27.5)	44/101 (43.6)	76/112 (67.9)
Missing data	4	0	0	1	3	0
Incarceration in the past 6 mo, No./total No. (%)^e^						
No	351/442 (79.4)	74/93 (79.6)	76/93 (81.7)	34/41 (82.9)	73/103 (70.9)	94/112 (83.9)
Yes	91/442 (20.6)	19/93 (20.4)	17/93 (18.3)	7/41 (17.1)	30/103 (29.1)	18/112 (16.1)
Missing	2	1	0	0	1	0
Ever taken MOUD						
No	164 (36.9)	90 (95.7)	27 (29.0)	13 (31.7)	30 (28.8)	4 (3.6)
Yes	280 (63.1)	4 (4.3)	66 (71.0)	28 (68.3)	74 (71.2)	108 (96.4)
Methadone treatment program in the past year, No./total No. (%)						
No	383/441 (86.8)	91/93 (97.8)	80/92 (87.0)	36/41 (87.8)	87/103 (84.5)	89/112 (79.5)
Yes	58/441 (13.2)	2/93 (2.2)	12/92 (13.0)	5/41 (12.2)	16/103 (15.5)	23/112 (20.5)
Missing, not known, or declined to answer	3	1	1	0	1	0
Engaged with harm reduction in the past year, No./total No. (%)^f^						
No	7/443 (1.6)	1/93 (1.1)	2/93 (2.2)	0	3/104 (2.9)	1/112 (0.9)
Yes	436/443 (98.4)	92/93 (98.9)	91/93 (97.8)	41/41 (100)	101/104 (97.1)	111/112 (99.1)
Missing, not known, or declined to answer	1	1	0	0	0	0

Abbreviation: MOUD, medications for opioid use disorder.

^a^ Four participants of White race and no reported ethnicity or unknown ethnicity were included in the non-Hispanic White group.

^b^ Includes the responses “American Indian or Alaska Native,” “Asian,” “Native Hawaiian or Other Pacific Islander,” “mixed race,” and “other (specify).”

^c^ Includes tiny home or transitional housing, hotel or motel, friend’s or family’s house, subsidized housing, pays for or owns private residence, or private residence—unspecified details.

^d^ Includes park, sidewalk, or other public property; abandoned building; public transportation station; car or personal vehicle; makeshift encampment or shelter; or unhoused—unspecified details.

^e^ Jail, prison, or other type of incarceration.

^f^ Included 2 questions on accessing clean needles and other drug use supplies.

Across all sites, approximately half of participants with housing data (203 of 440 [46.1%]) were unhoused at the time of enrollment, with higher rates in Philadelphia (76 of 112 [67.9%]) and New York City (56 of 94 [59.6%]). In contrast, Los Angeles had the lowest rate of homelessness, at 16 of 93 (17.2%). Recent incarceration was reported by 91 of 442 participants (20.6%) across all sites, with the highest rate in Houston (30 of 103 [29.1%]) and lowest in Philadelphia (18 of 112 [16.1%]). Nearly two-thirds of participants (280 [63.1%]) had ever taken MOUD, with 58 of 441 (13.2%) reporting being in a methadone treatment program within the past year and 436 of 443 (98.4%) reporting engaging with harm-reduction services in the year prior to enrollment (Table 1).

### Prevalence of Drugs Detected

Table 2 shows the prevalence of drugs detected across the study sites. Fentanyl was the most prevalent drug detected among all participants (414 [93.2%]). Use rates of cocaine (328 participants [73.9%]), methamphetamine or amphetamine (299 [67.3%]), and synthetic opiates (272 [61.3%]) were also high. Xylazine was detected in over half of participants (234 [52.7%]), and polysubstance use was detected among nearly all participants (421 [94.8%]), highlighting the complexity of toxicologic detection among participants.

**Table 2.** Baseline Drug Detection, Overall and by Site

Drug	Participants, No. (%)					
	Overall (N = 444)	New York City (n = 94)	Los Angeles (n = 93)	Washington, DC (n = 41)	Houston (n = 104)	Philadelphia (n = 112)
Amphetamine-type stimulants	299 (67.3)	25 (26.6)	87 (93.6)	12 (29.3)	84 (80.8)	91 (81.3)
Benzodiazepines	36 (8.1)	8 (8.5)	13 (14.0)	0	12 (11.5)	3 (2.7)
Buprenorphine	23 (5.2)	1 (1.1)	8 (8.6)	2 (4.9)	8 (7.7)	4 (3.6)
Cannabis	28 (6.3)	3 (3.2)	6 (6.5)	5 (12.2)	10 (9.6)	4 (3.6)
Cocaine	328 (73.9)	86 (91.5)	28 (30.1)	36 (87.8)	73 (70.2)	105 (93.8)
Fentanyl	414 (93.2)	91 (96.8)	87 (93.6)	40 (97.6)	85 (81.7)	111 (99.1)
Methadone	102 (23.0)	46 (48.9)	7 (7.5)	12 (29.3)	20 (19.2)	17 (15.2)
Opiates	272 (61.3)	80 (85.1)	42 (45.2)	21 (51.2)	81 (77.9)	48 (42.9)
Synthetic opioids	273 (61.5)	56 (59.6)	32 (34.4)	29 (70.7)	69 (66.3)	87 (77.7)
Xylazine	234 (52.7)	68 (72.3)	6 (6.5)	31 (75.6)	18 (17.3)	111 (99.1)
Polysubstance use^a^	421 (94.8)	87 (92.6)	89 (95.7)	39 (95.1)	95 (91.4)	111 (99.1)

^a^ Polysubstance drug detection is defined as liquid chromatography–high-resolution mass spectrometry detection of fentanyl or opioids with any stimulant, benzodiazepine, cocaine, and/or xylazine, recognizing that some combinations, such as fentanyl-xylazine, reflect coadulteration rather than intentional multiuse.

Notable differences in drug detection were observed by study site. Amphetamine-type stimulants were most prevalent in Los Angeles (87 of 93 [93.6%]) and Philadelphia (91 of 112 [81.3%]) but much lower in New York City (25 of 94 [26.6%]) and Washington, DC (12 of 41 [29.3%]). New York City (86 of 94 [91.5%]) and Philadelphia (105 of 112 [93.8%]) had the highest prevalence of cocaine detection, and Los Angeles (28 of 93 [30.1%]) had the lowest. Fentanyl detection was high across all cities, with the highest in Philadelphia (111 of 112 [99.1%]). Methadone detection was most prevalent in New York City (46 of 94 [48.9%]) and less common elsewhere (<30%). Xylazine was particularly prevalent in Philadelphia (111 of 112 [99.1%]), New York City (68 of 94 [72.3%]), and Washington, DC (31 of 41 [75.6%]), compared with low levels observed in Los Angeles (6 of 93 [6.5%]) and Houston (18 of 104 [17.3%]) (Table 2).

### Drug Detection by Age and Sex

No differences were noted by sex (eTable 1 in Supplement 1). A lower prevalence of methamphetamine or amphetamine was detected among participants younger than 30 years (31 of 49 [63.3%]) vs those aged 30 to 49 years (197 of 267 [73.8%]); difference, 16.4% (95% CI, 4.6%-28.3%; *P* = .007) (Table 3). A lower prevalence of stimulants was also detected among participants aged 50 years or older (71 of 128 [55.5%]) compared with those aged 30 to 49 years (difference, −8.8%; 95% CI, −17.5% to −0.04%; *P* = .049). For participants younger than 30 years, cocaine was detected in 28 of the 49 samples tested (57.1%), which was lower than among those aged 30 to 49 years (206 of 267 [77.2%]; difference, 17.0%; 95% CI, 4.8%-29.1%; *P* = .006) and those aged 50 years or older (94 of 128 [73.4%]; difference, 17.1%; 95% CI, 4.0%-30.2%; *P* = .01). Xylazine was more prevalent among participants aged 30 to 49 years (151 of 267 [56.6%]) compared with those younger than 30 years (19 of 49 [38.8%]; difference, 14.5%; 95% CI, 3.0%-26.0%; *P* = .01) and those aged 50 years or older (64 of 128 [50.0%]; difference, 13.5%; 95% CI, 1.4%-25.6%; *P* = .03). Methadone prevalence was highest in the eldest age group and ranged from 6 of 49 (12.2%) in participants younger than 30 years to 43 of 128 (33.6%) among those aged 50 years or older (difference, 16.2%; 95% CI, 3.0%-29.3%; *P* = .02).

**Table 3.** Comparisons of Drug Detection by Age^a^

Drug	Participants, No./total No. (%)	Age 30-49 y vs <30 y		Age ≥50 y vs < 30 y		Age ≥50 y vs 30-49 y	
		Estimated difference (95% CI), %^b^	*P* value	Estimated difference (95% CI), %^b^	*P* value	Estimated difference (95% CI), %^b^	*P* value
Amphetamine-type stimulants							
<30 y	31/49 (63.3)	16.4 (4.6 to 28.3)	.007	7.7 (−5.7 to 21.0)	.26	−8.8 (−17.5 to −0.04)	.049
30-49 y	197/267 (73.8)						
≥50 y	71/128 (55.5)						
Cocaine							
<30 y	28/49 (57.1)	17.0 (4.8 to 29.1)	.006	17.1 (4.0 to 30.2)	.01	0.1 (−7.5 to 7.7)	.98
30-49 y	206/267 (77.2)						
≥50 y	94/128 (73.4)						
Methadone							
<30 y	6/49 (12.2)	5.2 (−6.1 to 16.5)	.37	16.2 (3.0 to 29.3)	.02	10.9 (1.7 to 20.2)	.02
30-49 y	53/267 (19.9)						
≥50 y	43/128 (33.6)						
Xylazine							
<30 y	19/49 (38.8)	14.5 (3.0 to 26.0)	.01	13.5 (1.4 to 25.6)	.03	−1.1 (−7.9 to 5.7)	.76
30-49 y	151/267 (56.6)						
≥50 y	64/128 (50.0)						
Polysubstance use							
<30 y	43/49 (87.8)	9.8 (0.1 to 19.5)	.047	5.4 (−5.3 to 16.0)	.32	−4.4 (−9.3 to 0.4)	.07
30-49 y	260/267 (97.4)						
≥50 y	118/128 (92.2)						

^a^ Only significant results (*P* ≤ .05) for differences in drug detection are shown. Full output showing all drugs tested is in eTable 2 in Supplement 1.

^b^ Estimated differences were adjusted for site and therefore may differ from the direct group differences shown.

Polysubstance drugs were ubiquitous in all age groups, but use was most prevalent among those aged 30 to 49 years (260 of 267 [97.4%]) and least prevalent among participants younger than 30 years (43 of 49 [87.8%]; difference, 9.8%; 95% CI, 0.1%-19.5%; *P* = .047). In contrast, prevalence of benzodiazepines, buprenorphine, cannabis, fentanyl, opiates, and synthetic opioids was comparatively consistent across all age groups, with no notable differences (eTable 2 in Supplement 1).

### Drug Detection by Race and Ethnicity

White participants had higher prevalence of benzodiazepines compared with Hispanic or Latino participants (difference, 7.5%; 95% CI, 1.6%-13.3%; *P* = .01). Conversely, White participants had lower prevalence of opiate detection compared with Hispanic or Latino participants (difference, −14.3%; 95% CI, −25.3% to −3.3%; *P* = .01). Compared with White participants, Black participants had a lower prevalence of buprenorphine detection (difference, −6.8%; 95% CI, −13.4% to −0.1%; *P* = .050) (eTable 3 in Supplement 1). There were no differences in drug detection by race and ethnicity for other drug categories (eTable 3 in Supplement 1). Participants of other non-Hispanic race were excluded from this analysis due to small sample size.

### Drug Detection by Housing Status and Recent Incarceration

Drug detection by housing status and recent incarceration are shown in eTables 4 and 5 in Supplement 1. In brief, unhoused individuals had higher prevalence of cocaine detection compared with participants with housing (difference, 11.4%; 95% CI, 3.6%-19.2%; *P* = .004). Conversely, detection of benzodiazepines was lower among unhoused compared with housed individuals (difference, −5.7%; 95% CI, −10.9% to −0.5%; *P* = .03). Recently incarcerated individuals had a higher prevalence of amphetamine-type stimulant detection compared with those who had not been incarcerated (difference, 9.9%; 95% CI, 1.4%-18.5%; *P* = .02). We also observed lower prevalence of cannabis detection for those with no recent incarceration (difference, −7.8%; 95% CI, −10.6% to −5.0%; *P* < .01).

### Trends in Drug Detection Over Time Across the 5 Cities

Table 4 and the Figure show trends in amphetamine-type stimulants, fentanyl, xylazine, and polysubstance detection across the 5 study cities. Table 4 shows a modeled average change per 6-month period across the entire enrollment window, whereas the Figure shows the raw 6-month period-specific prevalence. of amphetamine-type stimulants, fentanyl, xylazine, and polysubstance detection across the 5 study cities. Although the sample size for Washington, DC, was small (n = 41), stimulants showed the most notable change over time, with an increase every 6 months of 15.0% (95% CI, 2.9%-27.1%; *P* = .02). While other sites showed mild fluctuations in stimulant detection, none were statistically significant. Fentanyl detection remained persistently high across all sites, typically near or above 90%, with no changes over time.

**Table 4.** Change in Amphetamine, Fentanyl, Xylazine, and Polysubstance Drug Detection Over 6-Month Intervals by Site^a^

Drug and site	Change over 6 mo of enrollment period (95 CI), %	*P* value
Amphetamine-type stimulants		
New York City	1.5 (−5.5 to 8.5)	.68
Los Angeles	1.7 (3.3 to 6.7)	.50
Washington, DC	15.0 (2.9 to 27.1)	.02
Houston	−3.0 (−8.8 to 2.7)	.30
Philadelphia	4.5 (−1.1 to 10.1)	.12
Fentanyl		
New York City	0.2 (−2.6 to 3.1)	.86
Los Angeles	−0.6 (−5.4 to 4.2)	.81
Washington, DC	−0.8 (−5.8 to 4.2)	.76
Houston	−4.3 (−10.0 to 1.4)	.14
Philadelphia^b^	−0.9 (−3.3 to 1.5)	.25
Xylazine		
New York City	10.3 (4.0 to 16.5)	.001
Los Angeles	0.6 (−4.2 to 5.4)	.81
Washington, DC	4.8 (−6.6 to 16.1)	.41
Houston	1.3 (−4.1 to 6.8)	.63
Philadelphia^b^	−0.9 (−3.3 to 1.5)	.25
Polysubstance		
New York City	5.1 (−0.6 to 10.8)	.08
Los Angeles	7.1 (−0.5 to 14.8)	.07
Washington, DC	3.0 (−3.6 to 9.6)	.37
Houston	1.6 (−2.4 to 5.6)	.44
Philadelphia^b^	−0.9 (−3.3 to 1.5)	.25

^a^ Change over time was estimated using period-specific proportions.

^b^ Philadelphia had nearly 100% fentanyl, xylazine, and polysubstance drug detection over the entire enrollment period.

**Figure.**
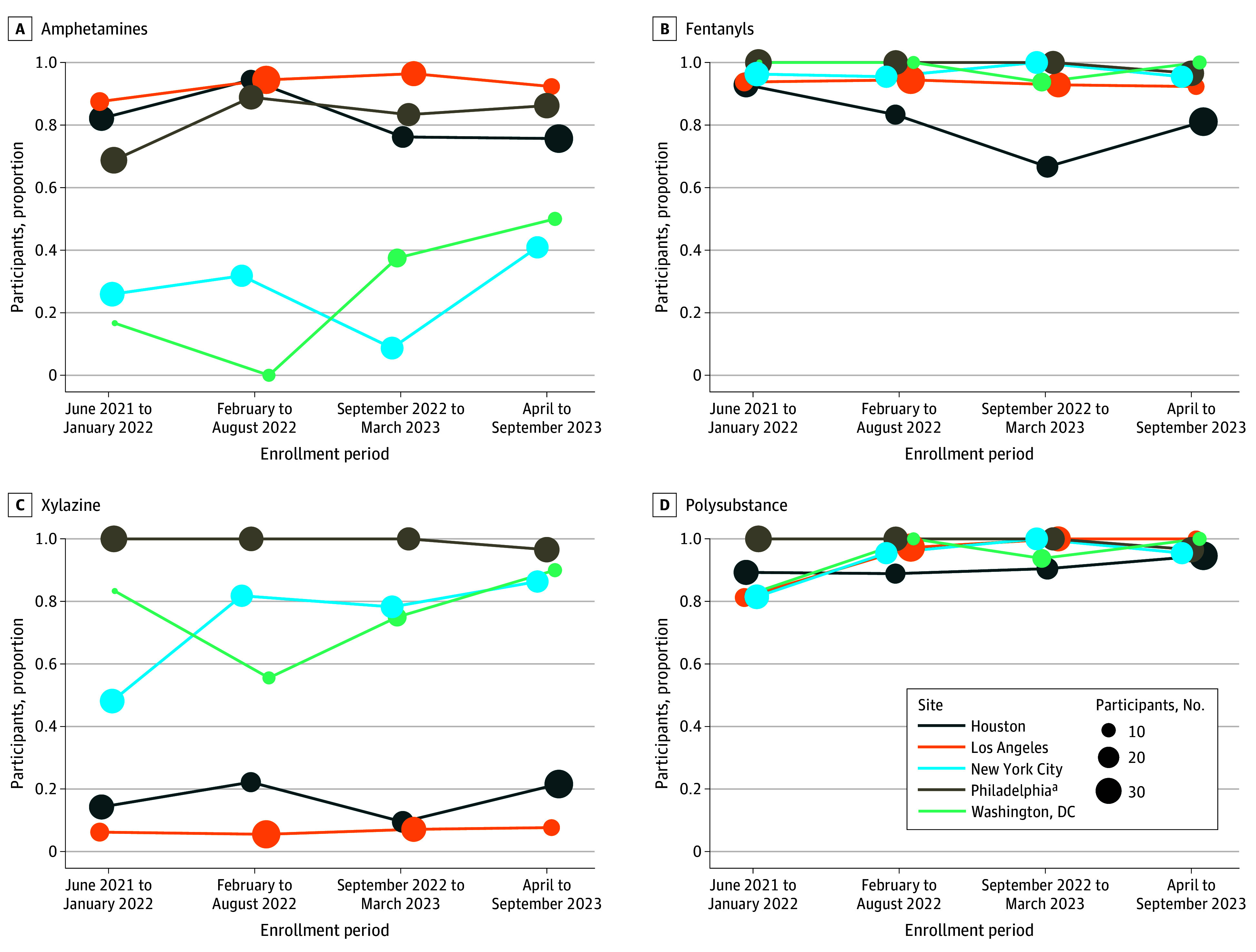
Frequency Graphs of Amphetamine, Fentanyl, Xylazine, and Polysubstance Drug Detection Over Enrollment Periods and by Site ^a^Philadelphia had nearly 100% fentanyl, xylazine, and polysubstance drug detection over the entire enrollment period. Change over time was estimated using period-specific proportions.

New York City showed an upward trend in xylazine detection, with an increase of 10.3% (95% CI, 4.0%-16.5%) every 6 months (*P* = .001). Other sites showed no meaningful shifts in xylazine detection, with Philadelphia participants having nearly 100% xylazine detection and Los Angeles and Houston having low detection across all enrollment periods. Polysubstance detection remained high across all sites throughout the enrollment period, with prevalence typically exceeding 90%. While no city showed statistically significant changes in the detection of polysubstance drugs over time, New York City and Los Angeles exhibited upward trends.

## Discussion

This study offers a unique and timely contribution by mapping the toxicologic illicit drug detection across 5 diverse US cities participating in the HPTN 094 study. Despite regional differences in prevalence and types of drugs, fentanyl and polysubstance drug prevalence remained uniformly high, underscoring drug penetrance across urban US drug markets at the time of study enrollment. We also found increases in amphetamine-type stimulant detection in Washington, DC, xylazine in New York City, and polysubstance drugs in New York City and Los Angeles, highlighting important local shifts in drug trends that may inform targeted public health responses. We also note that the rise in polysubstance detection may be from adulteration within the unregulated supply rather than intentional co-use.

Using baseline data over time, a notable finding from this study was the apparent east-to-west diffusion of xylazine, which was initially concentrated in northeastern cities such as New York, Philadelphia, and Washington, DC, but showed signs of westward spread over time.^24^ The temporal rise in xylazine detection in these eastern cities, coupled with its lower baseline prevalence in western sites like Los Angeles and Houston, suggests that western regions may be in earlier phases of exposure. This geographic progression mirrors historical patterns seen with fentanyl and other synthetic drugs, where East Coast markets often serve as early indicators of broader national shifts.^17^ Such trends emphasize the importance of proactive drug surveillance systems capable of detecting and responding to emergent substances before they become entrenched in local drug supplies. The implications for public health and clinical response are important for locations that have not yet experienced high xylazine penetration and may still have a window of opportunity for targeted harm reduction, community education, and health care professional training on its unique toxicologic profile. More broadly, this east-to-west dynamic underscores the value of using geographically granular, time-sensitive data to forecast drug trends and prevent reactive policymaking.

Furthermore, our findings showed that xylazine positivity was commonly observed in participants aged 30 to 49 years, consistent with published reports indicating that xylazine use is more prevalent among younger individuals.^25^ The CDC and recent toxicology analyses have shown a growing prevalence of xylazine in overdose deaths among individuals aged in their 30s and 40s, particularly in regions heavily impacted by fentanyl-adulterated drug supplies.^24,26^ This demographic trend may reflect the intersection of younger age groups’ exposure to synthetic drug markets and polysubstance use patterns, suggesting an urgent need for targeted harm-reduction strategies that account for xylazine’s sedative effects and its combination with opioids in this population. The rise in amphetamine-type stimulant detection in Washington, DC, and xylazine in New York City aligns with national alerts and provides site-specific evidence useful for targeted interventions. Age-related differences in stimulant, methadone, and xylazine detection suggest distinct drug risk profiles.

When examining differences by housing status, unhoused individuals had higher prevalence of cocaine detection but lower benzodiazepine detection compared with housed individuals, possibly indicating patterns shaped by market access or preference. Ethnographic research also suggests that stimulant use among unhoused individuals may serve functional purposes such as maintaining alertness for safety in vulnerable environments, which aligns with our finding of higher cocaine detection among unhoused participants.^27^ People with recent history of incarceration had higher prevalence of stimulant detection but lower cannabis detection. These results call for regionally tailored harm-reduction strategies and drug supply monitoring systems that include real-time drug testing.^28^

The individuals reached in this study faced numerous challenges, including homelessness, lack of access to drug treatment and other health or social services, and a history of incarceration. The HPTN 094 qualitative findings revealed that participants represent a severely stigmatized population that is often excluded from drug treatment systems and other health care systems.^29,30^ Thus, the integrated approach within the mobile health unit used in the HPTN 094 study was critical for reaching this hard-to-reach key population.^30,31^

### Strengths and Limitations

This study has several strengths. This study contributes participant-level, biomarker-verified drug detection data on people who inject drugs and are not engaged in medical care across 5 US cities, filling a gap left by population-based surveillance and drug-checking programs that rarely capture this subgroup.^32^ The study successfully reached a hard-to-reach population that is often disconnected from drug treatment services and health care and is underrepresented in research. We linked biomarker (LC-HRMS)–verified drug detection to sociodemographic and structural (housing status, incarceration) vulnerabilities, distinguishing it from aggregate drug-checking data.

This study also has limitations. First, although HPTN 094 was a randomized clinical trial targeting a very difficult-to-reach population of individuals who inject drugs and have opioid use disorders, random sampling was not feasible. Because all 5 sites were large urban centers and enrollment was restricted to people who inject drugs, findings may not reflect drug use patterns among rural populations or individuals who smoke or inhale drugs—the fastest-growing route of use nationally. As a result, the generalizability of our findings to the broader population of people who inject drugs is limited. Second, although HPTN 094 collected individual-level biomarker data over time, this analysis only used baseline biomarker data to assess what drugs were detected in the urine of people who inject drugs enrolled in HPTN 094 over calendar time. This analysis relied on a single-time-point toxicology assessment, which constrained our ability to determine causal relationships or observe changes in individual drug use patterns over time. Third, power for subgroup analyses was limited: some site-specific comparisons, such as those from Washington, DC (n = 41), may be underpowered due to small sample sizes, which limits the reliability and interpretability of those localized findings. Fourth, due to sample size constraints, we could not adequately adjust for potential confounders within each enrollment period to avoid unstable estimates and overfitting. The changes in drug detection over time should be interpreted as crude estimates, reflecting a combination of both compositional and temporal effects. Fifth, analyses presented did not account for possible correlations of individuals within communities. However, enrollments occurred across several locations within each study site, and estimated intraclass correlations were generally small. Sensitivity analyses were performed using logistic regression with exchangeable correlation within sites and yielded no significant changes to the analyses presented. Overall, our findings highlight variations in toxicologic detection by region and sociodemographic characteristics and changes in drug detection over time, which are important for informing tailored harm-reduction strategies.

## Conclusions

This cross-sectional analysis of HPTN 094 data provided robust, geographically diverse data showing that fentanyl and polysubstance drugs continue to dominate the urban drug landscape. These findings offer a timely, comparative overview of individual-level illicit drug detection among non–medically engaged people who inject drugs and have opioid use disorders across 5 US cities, including a newly emerging illicit drug supply. The study’s multisite design was both novel and essential for understanding current injection drug trends in the US and implications for treatment services targeting this population. Despite national narratives emphasizing a shift toward drug inhalation, injection-related overdose deaths remain high, particularly due to rising use of dangerous combinations involving xylazine, stimulants, and polydrug use with fentanyl. These findings underscore an urgent need for real-time surveillance of the illicit drug supply to rapidly identify emerging threats. Furthermore, integrating harm-reduction and treatment approaches that address both substance use and underlying social determinants of health is essential to reduce overdose mortality, mitigate health disparities, and strengthen public health responses to evolving drug epidemics.

## Notes

Supplement 1. **eTable 1.** Comparison of Drug Detection by Sex at Birth **eTable 2.** Comparison of Drug Detection by Age Group **eTable 3.** Comparison of Drug Detection by Race and Ethnicity **eTable 4.** Comparison of Drug Detection Overall, by Housing Status **eTable 5.** Comparison of Drug Detection Overall, by Incarceration in Last 6 Months **eAppendix.** Information on the LC-HRMS Testing

Supplement 2.
HPTN 094 Study Team

Supplement 3.
Data Sharing Statement

## References

[1] Bradley H, Hall EW, Asher A, . Estimated number of people who inject drugs in the United States. Clin Infect Dis. 2023;76(1):96-102. doi:10.1093/cid/ciac543 35791261 PMC10202436

[2] Friedman J, Shover CL. Charting the fourth wave: geographic, temporal, race/ethnicity and demographic trends in polysubstance fentanyl overdose deaths in the United States, 2010-2021. Addiction. 2023;118(12):2477-2485. doi:10.1111/add.16318 37705148

[3] Garnett MF, Miniño AM. Drug overdose deaths in the United States, 2003-2023. National Center for Health Statistics data brief No. 522. December 2024. Accessed September 7, 2025. https://www.cdc.gov/nchs/products/databriefs/db522.htm

[4] Berk J, South AM, Martin M, . Medication for opioid use disorder service delivery in carceral facilities: update and summary report. Health Justice. 2025;13(1):8. doi:10.1186/s40352-025-00317-9 39891797 PMC11786385

[5] Merrall EL, Kariminia A, Binswanger IA, . Meta-analysis of drug-related deaths soon after release from prison. Addiction. 2010;105(9):1545-1554. doi:10.1111/j.1360-0443.2010.02990.x 20579009 PMC2955973

[6] Binswanger IA, Stern MF, Deyo RA, . Release from prison—a high risk of death for former inmates. N Engl J Med. 2007;356(2):157-165. doi:10.1056/NEJMsa064115 17215533 PMC2836121

[7] Lee JD, McDonald R, Grossman E, . Opioid treatment at release from jail using extended-release naltrexone: a pilot proof-of-concept randomized effectiveness trial. Addiction. 2015;110(6):1008-1014. doi:10.1111/add.12894 25703440

[8] Ford JD, Trestman RL, Wiesbrock VH, Zhang W. Validation of a brief screening instrument for identifying psychiatric disorders among newly incarcerated adults. Psychiatr Serv. 2009;60(6):842-846. doi:10.1176/ps.2009.60.6.842 19487358

[9] Yamamoto A, Needleman J, Gelberg L, Kominski G, Shoptaw S, Tsugawa Y. Association between homelessness and opioid overdose and opioid-related hospital admissions/emergency department visits. Soc Sci Med. 2019;242:112585. doi:10.1016/j.socscimed.2019.112585 31634808 PMC7023863

[10] Cano M, Oh S. State-level homelessness and drug overdose mortality: evidence from US panel data. Drug Alcohol Depend. 2023;250:110910. doi:10.1016/j.drugalcdep.2023.110910 37535991 PMC10530113

[11] Bauer LK, Brody JK, León C, Baggett TP. Characteristics of homeless adults who died of drug overdose: a retrospective record review. J Health Care Poor Underserved. 2016;27(2):846-859. doi:10.1353/hpu.2016.0075 27180712 PMC4911892

[12] Doran KM, Misa EJ, Shah NR. Housing as health care: New York’s boundary-crossing experiment. Obstet Gynecol Surv. 2014;69(4):195-197. doi:10.1097/01.ogx.0000446906.02266.3c 24350949

[13] Levitt A, Mermin J, Jones CM, See I, Butler JC. Infectious diseases and injection drug use: public health burden and response. J Infect Dis. 2020;222(suppl 5):S213-S217. doi:10.1093/infdis/jiaa432 32877539

[14] Hall EW, Sullivan PS, Bradley H. Estimated number of injection-involved overdose deaths in US states from 2000 to 2020: secondary analysis of surveillance data. JMIR Public Health Surveill. 2024;10:e49527. doi:10.2196/49527 38578676 PMC11031697

[15] Girardi E, Zaccarelli M, Tossini G, Puro V, Narciso P, Visco G. Hepatitis C virus infection in intravenous drug users: prevalence and risk factors. Scand J Infect Dis. 1990;22(6):751-752. doi:10.3109/00365549009027133 2178279

[16] US overdose deaths decrease almost 27% in 2024. News release. National Center for Health Statistics, Centers for Disease Control and Prevention. May 14, 2025. Accessed September 7, 2025. https://www.cdc.gov/nchs/pressroom/releases/20250514.html

[17] Ciccarone D. The rise of illicit fentanyls, stimulants and the fourth wave of the opioid overdose crisis. Curr Opin Psychiatry. 2021;34(4):344-350. doi:10.1097/YCO.0000000000000717 33965972 PMC8154745

[18] Centers for Disease Control and Prevention. Polysubstance overdose. May 8, 2024. Accessed September 6, 2025. https://www.cdc.gov/overdose-prevention/about/polysubstance-overdose.html

[19] Jenkins RA, Whitney BM, Nance RM, ; Rural Opioid Initiative. The Rural Opioid Initiative Consortium description: providing evidence to understand the fourth wave of the opioid crisis. Addict Sci Clin Pract. 2022;17(1):38. doi:10.1186/s13722-022-00322-5 35883197 PMC9321271

[20] Kral AH, Lambdin BH, Browne EN, . Transition from injecting opioids to smoking fentanyl in San Francisco, California. Drug Alcohol Depend. 2021;227:109003. doi:10.1016/j.drugalcdep.2021.109003 34482046 PMC10790652

[21] Reid MC, Oliphant-Wells T, Moreno C, . High levels of interest in access to free safer smoking equipment to reduce injection frequency among people who inject drugs in Seattle, Washington. Drug Alcohol Depend Rep. 2023;7:100163. doi:10.1016/j.dadr.2023.100163 37214756 PMC10193167

[22] Goodman-Meza D, Shoptaw S, Hanscom B, ; HPTN 094 Study Team. Delivering integrated strategies from a mobile unit to address the intertwining epidemics of HIV and addiction in people who inject drugs: the HPTN 094 randomized controlled trial protocol (the INTEGRA Study). Trials. 2024;25(1):124. doi:10.1186/s13063-023-07899-5 38360750 PMC10870682

[23] von Elm E, Altman DG, Egger M, Pocock SJ, Gøtzsche PC, Vandenbroucke JP; STROBE Initiative. The Strengthening the Reporting of Observational Studies in Epidemiology (STROBE) statement: guidelines for reporting observational studies. Lancet. 2007;370(9596):1453-1457. doi:10.1016/S0140-6736(07)61602-X 18064739

[24] US Drug Enforcement Administration. The growing threat of xylazine and its mixture with illicit drugs. October 2022. Accessed September 6, 2025. https://www.dea.gov/sites/default/files/2022-12/The%20Growing%20Threat%20of%20Xylazine%20and%20its%20Mixture%20with%20Illicit%20Drugs.pdf

[25] Jiang X, Connolly S, Strahan AE, . Reported xylazine use among adults aged ≥18 years evaluated for substance use treatment—United States, July 2022-September 2023. MMWR Morb Mortal Wkly Rep. 2024;73(26):594-599. doi:10.15585/mmwr.mm7326a2 38959171 PMC11221633

[26] Kariisa M, Patel P, Smith H, Bitting J. Notes from the field: xylazine detection and involvement in drug overdose deaths—United States, 2019. MMWR Morb Mortal Wkly Rep. 2021;70(37):1300-1302. doi:10.15585/mmwr.mm7037a4 34529640 PMC8445380

[27] McKenna SA. “We’re supposed to be asleep?” vigilance, paranoia, and the alert methamphetamine user. Anthropol Conscious. 2013;24(2):172-190. doi:10.1111/anoc.12012 26366049 PMC4563997

[28] Maghsoudi N, Tanguay J, Scarfone K, . Drug checking services for people who use drugs: a systematic review. Addiction. 2022;117(3):532-544. doi:10.1111/add.15734 34729849 PMC9299873

[29] Perez-Brumer A, Schmidt R, Kennedy R, . Centering autonomy and choice to support oral PrEP utilization among people who inject drugs: qualitative lessons from HPTN 094 INTEGRA. Addict Sci Clin Pract. 2024;19(1):92. doi:10.1186/s13722-024-00520-3 39696609 PMC11653907

[30] Smith LR, Perez-Brumer A, Nicholls M, ; HPTN 094 study protocol team. A data-driven approach to implementing the HPTN 094 complex intervention INTEGRA in local communities. Implement Sci. 2024;19(1):39. doi:10.1186/s13012-024-01363-x 38831415 PMC11149235

[31] Shoptaw S, El-Bassel N, Andrew P, . HPTN 094 Study Team. Preliminary efficacy for HPTN 094: 26-week RCT of integrated strategies for people who inject drugs. Presented at: Conference on Retroviruses and Opportunistic Infections; March 9-12, 2025; San Francisco, California. Accessed September 6, 2025. https://www.hptn.org/sites/default/files/inline-files/HPTN%20094%20CROI%202025%20-%203-8-2025.pdf

[32] Cottler LB, Goldberger BA, Nixon SJ, . Introducing NIDA’s new national drug early warning system. Drug Alcohol Depend. 2020;217:108286. doi:10.1016/j.drugalcdep.2020.108286 32979739 PMC7489265

