# Supplementary material for: Prevalence of Illicit Drug Detection in 5 US Cities Among Out-of-Treatment People Who Inject Drugs
Source: JAMA Netw Open. 2026 Feb 5;9(2):e2555882. doi: 10.1001/jamanetworkopen.2025.55882 (PMC12878425; doi:10.1001/jamanetworkopen.2025.55882)
Supplement: Supplement 1. — eTable 1. Comparison of Drug Detection by Sex at Birth eTable 2. Comparison of Drug Detection by Age Group eTable 3. Comparison of Drug Detection by Race and Ethnicity eTable 4. Comparison of Drug Detection Overall, by Housing Status eTable 5. Comparison of Drug Detection Overall, by Incarceration in Last 6 Months eAppendix. Information on the LC-HRMS Testing [file jamanetwopen-e2555882-s001.pdf]

Supplementary Online Content

El-Bassel N, Shoptaw S, Skalland T, et al; HPTN 094 Study Team. Prevalence of illicit drug detection in 5 US cities among out-of-treatment people who inject drugs. *JAMA Netw Open*. 2026;9(2):e2555882. doi:10.1001/jamanetworkopen.2025.55882

eTable 1. Comparison of Drug Detection by Sex at Birth

eTable 2. Comparison of Drug Detection by Age Group

eTable 3. Comparison of Drug Detection by Race and Ethnicity

eTable 4. Comparison of Drug Detection Overall, by Housing Status

eTable 5. Comparison of Drug Detection Overall, by Incarceration in Last 6 Months

eAppendix. Information on the LC-HRMS Testing

This supplementary material has been provided by the authors to give readers additional information about their work.

© 2026 El-Bassel N et al. *JAMA Network Open*.

eTable 1. Comparison of drug detection by sex at birth

	Sex at Birth		Comparisons:<br>Estimated Difference, 95% CI, p-value
**Drug category**	**Female**	**Male**	**Female vs Male**
Amphetamine-type Stimulants	95/141 (67.4%)	204/303 (67.3%)	3.3% (-4.4%, 11.1%) *p*=0.40
Benzodiazepines	11/141 (7.8%)	25/303 (8.3%)	0.3% (-5.1%, 5.6%) *p*=0.93
Buprenorphine	6/141 (4.3%)	17/303 (5.6%)	1.3% (-2.9%, 5.5%) *p*=0.53
Cannabis	5/141 (3.6%)	23/303 (7.6%)	3.9% (-0.4%, 8.2%) *p*=0.08
Cocaine(s)	107/141 (75.9%)	221/303 (72.9%)	-1.0% (-8.4%, 6.3%) *p*=0.78
Fentanyls	130/141 (92.2%)	284/303 (93.7%)	2.5% (-3.7%, 6.3%) *p*=0.60
Methadone	26/141 (18.4%)	76/303 (25.1%)	5.6% (-2.1%, 13.3%) *p*=0.16
Opiates	90/141 (63.8%)	182/303 (60.1%)	-4.4% (-13.4%, 4.6%) *p*=0.34
Synthetic Opioids	91/141 (64.5%)	182/303 (60.1)	-2.5% (-11.7%, 6.8%) *p*=0.61
Xylazine	73/141 (51.8%)	161/303 (53.1%)	4.2% (-2.5%, 11.0%) *p*=0.22

© 2026 El-Bassel N et al. *JAMA Network Open*.

Polysubstance Use	135/141 (95.7%)	286/303 (94.4%)	-1.1% (-5.4%, 3.1%) *p*=0.60

Estimated differences are adjusted for site and therefore may differ from the direct group differences seen in the table.

© 2026 El-Bassel N et al. *JAMA Network Open*.

eTable 2. Comparison of drug detection by age group

	Age group			Comparisons: Estimated Difference, 95% CI, p-value		
**Drug category**	**<30**	**30-49**	**50+**	**<30 vs 30-49**	**<30 vs 50+**	**30-49 vs 50+**
Amphetamine-type Stimulants	31/49 (63.3%)	197/267 (73.8%)	71/128 (55.5%)	16.4% (4.6%, 28.3%) *p*=0.007	7.7% (-5.7%, 21.0%) *p*=0.26	-8.8% (-17.5%, 0.00) *p*=0.049
Benzodiazepines	5/49 (10.2%)	23/267 (8.6%)	8/128 (6.3%)	-1.3% (-10.3%, 7.6%) *p*=0.77	-4.0% (-13.4%, 5.3%) *p*=0.40	-2.7% (-8.0%, 2.6%) *p*=0.32
Buprenorphine	2/49 (4.1%)	16/267 (6.0%)	5/128 (3.9%)	2.5% (-3.2%, 8.2%) *p*=0.38	0.3% (-5.7%, 6.3%) *p*=0.93	-2.3% (-6.8%, 2.3%) *p*=0.33
Cannabis	4/49 (8.2%)	18/267 (6.7%)	6/128 (4.7%)	-0.5% (-8.4%, 7.4%) *p*=0.90	-3.5% (-11.6%, 4.6%) *p*=0.39	-3.0% (-7.7%, 1.7%) *p*=0.21
Cocaine(s)	28/49 (57.1%)	206/267 (77.2%)	94/128 (73.4%)	17.0% (4.8%, 29.1%) *p*=0.006	17.1% (4.0%, 30.2%) *p*=0.01	0.1% (-7.5%, 7.7%) *p*=0.98
Fentanyls	46/49 (93.9%)	251/267 (94.0%)	117/128 (91.4%)	-1.3% (-7.1%, 4.5%) *p*=0.65	-4.7% (-12.0%, 2.5%) *p*=0.20	-3.4% (-9.2%, 2.4%) *p*=0.25
Methadone	6/49 (12.2%)	53/267 (19.9%)	43/128 (33.6%)	5.2% (-6.1%, 16.5%) *p*=0.37	16.2% (3.0%, 29.3%) *p*=0.02	10.9% (1.7%, 20.2%) *p*=0.02
Opiates	28/49 (57.1%)	164/267 (61.4%)	80/128 (62.5%)	3.6% (-10.1%, 17.4%) *p*=0.61	2.5% (-12.8%, 17.8%) *p*=0.75	-1.1% (-11.1%, 8.9%) *p*=0.83
Synthetic Opioids	30/49 (61.2%)	167/267 (62.5%)	76/128 (59.4%)	2.5% (-12.0%, 16.9%) *p*=0.74	3.8% (-11.9%, 19.6%) *p*=0.64	1.4% (-8.6%, 11.3%) *p*=0.79
Xylazine	19/49 (38.8%)	151/267 (56.6%)	64/128 (50.0%)	14.5% (3.0%, 26.0%) *p*=0.01	13.5% (1.4%, 25.6%) *p*=0.03	-1.1% (-7.9%, 5.7%) *p*=0.76

© 2026 El-Bassel N et al. *JAMA Network Open*.

Polysubstance Use	43/49 (87.8%)	260/267 (97.4%)	118/128 (92.2%)	9.8% (0.1%, 19.5%) *p*=0.047	5.4% (-5.3%, 16.0%) *p*=0.32	-4.4% (-9.3%, 0.4%) *p*=0.07

Estimated differences are adjusted for site and therefore may differ from the direct group differences seen in the table.

© 2026 El-Bassel N et al. *JAMA Network Open*.

eTable 3. Comparison of drug detection by race and ethnicity

	Race/Ethnicity			Comparisons: Estimated Difference, 95% CI, p-value		
**Drug category**	**Hispanic /Latino**	**Non-Hispanic White**	**Non-Hispanic Black**	**Hispanic vs Non-Hispanic White**	**Hispanic vs Non-Hispanic Black**	**Non-Hispanic White vs Non-Hispanic Black**
Amphetamine-type Stimulants	96/145 (66.2%)	160/199 (80.4%)	34/83 (41.0%)	1.9% (-8.1%, 12.0) *p*=0.71	-8.0% (-21.0%, 5.0%) *p*=0.23	-9.9% (-23.2%, 3.4%) *p*=0.14
Benzodiazepines	9/145 (6.2%)	22/199 (11.1%)	5/83 (6.0%)	7.5% (1.6%, 13.3%) *p*=0.01	5.5% (-3.5%, 14.5%) *p*=0.23	-1.9% (-12.1%, 8.2%) *p*=0.71
Buprenorphine	6/145 (4.1%)	14/199 (7.0%)	1/83 (1.2%)	3.6% (-2.2%, 9.3%) *p*=0.22	-3.2% (-8.1%, 1.7%) *p*=0.20	-6.8% (-13.4%, -0.1%) *p*=0.05
Cannabis	8/145 (5.5%)	11/199 (5.5%)	8/83 (9.6%)	-0.4% (-5.8%, 5.0%) *p*=0.89	2.7% (-6.4%, 11.9%) *p*=0.56	3.1% (-5.9%, 12.2%) *p*=0.50
Cocaine(s)	95/145 (65.5%)	147/199 (73.9%)	74/83 (89.2%)	-1.5% (-9.8%, 6.9%) *p*=0.73	6.9% (-5.8%, 19.6%) *p*=0.29	8.4% (-4.5%, 21.2%) *p*=0.20
Fentanyls	136/145 (93.8%)	187/199 (94.0%)	76/83 (91.6%)	0.70% (-4.0%, 5.4%) *p*=0.77	-9.9% (-21.0%, 1.3%) *p*=0.08	-10.6% (-21.6%, 0.4%) *p*=0.06
Methadone	38/145 (26.2%)	31/199 (15.6%)	29/83 (34.9%)	-4.2% (-13.9%, 5.6%) *p*=0.40	4.5% (-7.9%, 17.0%) *p*=0.48	8.7% (-4.0%, 21.4%) *p*=0.18
Opiates	104/145 (71.7%)	105/199 (52.8%)	53/83 (63.9%)	-14.3% (-25.3%, -3.3%) *p*=0.01	-7.0% (-21.9%, 8.0%) *p*=0.36	7.4% (-7.6%, 22.4%) *p*=0.33
Synthetic Opioids	82/145 (56.6%)	122/199 (61.3%)	59/83 (71.1%)	-9.2% (-20.1%, 1.6%) *p*=0.09	4.8% (-9.1%, 18.7%) *p*=0.50	14.0% (-0.5%, 28.6%) *p*=0.06

© 2026 El-Bassel N et al. *JAMA Network Open*.

Xylazine	62/145 (42.8%)	107/199 (53.8%)	54/83 (65.1%)	-4.4% (-12.4%, 3.6%) *p*=0.28	-6.7% (-15.4%, 2.0%) *p*=0.13	-2.3% (-12.3%, 7.6%) *p*=0.65
Polysubstance Use	139/145 (95.9%)	190/199 (95.5%)	76/83 (91.6%)	-1.8% (-6.3%, 2.7%) *p*=0.43	-6.0% (-14.2%, 2.2%) *p*=0.15	-4.2% (-13.1%, 4.7%) *p*=0.36

Estimated differences are adjusted for site and therefore may differ from the direct group differences seen in the table.

© 2026 El-Bassel N et al. *JAMA Network Open*.

eTable 4. Comparison of drug detection overall, by housing status

	Housing Status		Comparisons: Estimated Difference, 95% CI, p-value
**Drug category**	**Housed**	**Unhoused**	**Housed vs Unhoused**
Amphetamine-type Stimulants	160/237 (67.5%)	136/203 (67.0%)	5.9% (-1.7%, 13.5%) p=0.13
Benzodiazepines	27/237 (11.4%)	9/203 (4.4%)	-5.7% (-10.9%, -0.5%) p=0.03
Buprenorphine	15/237 (6.3%)	6/203 (3.0%)	-2.1% (-6.3%, 2.1%) p=0.33
Cannabis	16/237 (6.8%)	11/203 (5.4%)	0.2% (-4.7%, 5.1%) p=0.94
Cocaine(s)	147/237 (62.0%)	178/203 (87.7%)	11.4% (3.6%, 19.2%) p=0.004
Fentanyls	219/237 (92.4%)	191/203 (94.1%)	0.05% (-4.8%, 4.9%) p=0.98
Methadone	56/237 (23.6%)	46/203 (22.7%)	-6.3% (-13.4%, 1.5%) p=0.11
Opiates	141/237 (59.5%)	129/203 (63.5%)	1.5% (-7.8%, 10.8%) p=0.75
Synthetic Opioids	136/237 (57.4%)	137/203 (67.5%)	1.1% (-8.2%, 10.5%) p=0.81
Xylazine	96/237 (40.5%)	137/203 (67.5%)	4.1% (-2.5%, 10.7%) p=0.22

© 2026 El-Bassel N et al. *JAMA Network Open*.

Polysubstance Use	223/237 (94.1%)	194/203 (95.6%)	1.0% (-3.3%, 5.4%) *p*=0.64

Estimated differences are adjusted for site and therefore may differ from the direct group differences seen in the table.

© 2026 El-Bassel N et al. *JAMA Network Open*.

eTable 5. Comparison of drug detection overall, by incarceration in last 6 months

	Incarceration in last 6 months?		Comparisons: Estimated Difference, 95% CI, p-value
**Drug category**	**No**	**Yes**	**No vs Yes**
Amphetamine-type Stimulants	229/351 (65.2%)	69/91 (75.8%)	9.9% (1.4%, 18.5%) p=0.02
Benzodiazepines	30/351 (8.6%)	6/91 (6.6%)	-2.9% (-9.6%, 3.8%) p=0.40
Buprenorphine	20/351 (5.7%)	3/91 (3.3%)	-3.2% (-9.3%, 2.9%) p=0.31
Cannabis[1]	27/351 (7.7%)	0/91 (0.0%)	--7.8% (-10.6%, -5.0%) p<0.01
Cocaine(s)	258/351 (73.5%)	68/91 (74.7%)	2.1% (-6.3%, 10.4%) p=0.63
Fentanyls	328/351 (93.4%)	84/91 (92.3%)	0.7% (-4.7%, 6.1%) *p*=0.79
Methadone	83/351 (23.6%)	19/91 (20.9%)	-3.2% (-12.6%, 6.1%) *p*=0.50
Opiates	211/351 (60.1%)	59/91 (64.8%)	0.8% (-9.8%, 11.5%) *p*=0.88
Synthetic Opioids	216/351 (61.5%)	56/91 (61.5%)	0.0% (-10.7%, 10.6%) *p*=0.99
Xylazine	191/351 (54.4%)	43/91 (47.3%)	-0.5% (-7.9%, 6.9%) *p*=0.90

© 2026 El-Bassel N et al. *JAMA Network Open*.

Polysubstance Use	333/351 (94.9%)	86/91 (94.5%)	0.4% (-4.6%, 5.4%) *p*=0.88

Estimated differences are adjusted for site and therefore may differ from the direct group differences seen in the table.
[1]The generalized risk difference model did not converge, therefore we used a normal linear regression model with proportions.

© 2026 El-Bassel N et al. *JAMA Network Open*.

eAppendix. Information on the LC-HRMS testing

Positive results were defined as any presence above the cutoff/ limit of detection (LOD). For a compound to be identified positively, it must have the appropriate high-resolution mass for the precursor ion within 5 ppm, must have the appropriate chromatographic retention time (RT) within +/- 0.2 min of the expected RT, and must have a Library Score comparing experimental spectra versus library spectra for the MS2 (fragments spectrum) of greater than 70 – all criteria must be met for a positive result. The exceptions for this are buprenorphine-glucuronide and hydrocodone, which have alternate criteria defined. All spectra for each drug are manually reviewed by 2 analysts, and any equivocal results are adjudicated by the Lab Director.

Concentration cutoffs (LOD) were determined for each drug in the panel by analysis of 5 replicates in decreasing concentrations for each analyte; the LOD was defined as the concentration for which at least 4 of the 5 replicates yielded positive results.

Selectivity of the method was established by analysis of 5 different blank urine specimens submitted for routine toxicology screening that were determined to be blank by our reference/comparator toxicology screening method. No positive matches were seen in these blank urines. Carryover was also assessed, and any specimens with a positive result where the previous specimen had a positive result at or above the carryover limit were re-analyzed.

For accuracy/concordance relative to the reference method, 50 specimens were analyzed and results compared between methods. The positive predictive value was calculated at 97%, the negative predictive value calculated at 99%, and the diagnostic efficiency was calculated at 99%.

© 2026 El-Bassel N et al. *JAMA Network Open*.
