# Supplementary material for: Prevalence of Illicit Drug Detection in 5 US Cities Among Out-of-Treatment People Who Inject Drugs
Source: JAMA Netw Open. 2026 Feb 5;9(2):e2555882. doi: 10.1001/jamanetworkopen.2025.55882 (PMC12878425; doi:10.1001/jamanetworkopen.2025.55882)
Supplement: Supplement 2. — HPTN 094 Study Team [file jamanetwopen-e2555882-s002.pdf]

Supplemental Online Content: Nonauthor Collaborators
*First name, last name, and suffix (if applicable) are required and will appear in PubMed.

*Group Name(s): HPTN 094 Study Team							
***First Name and Middle Initial(s)**	***Last Name**	***Suffix (eg, Jr, III)**	Academic Degrees	Institution	Location (city, state/province, country)	Role or Contribution, eg, chair, principal investigator	Group (if more than 1 Group listed in the byline) and/or Subgroup (eg, Steering Committee)
Quiana	Allen		FNP-C				
Rashaunna	Redd		DNP				
David	Metzger		PhD				
Usiel	Gutierrez		LVN				
Manya	Magnus		PhD				
Marc	Siegel		MD				
