# Supplementary material for: Prevalence of Illicit Drug Detection in 5 US Cities Among Out-of-Treatment People Who Inject Drugs
Source: JAMA Netw Open. 2026 Feb 5;9(2):e2555882. doi: 10.1001/jamanetworkopen.2025.55882 (PMC12878425; doi:10.1001/jamanetworkopen.2025.55882)
Supplement: Supplement 3. — Data Sharing Statement [file jamanetwopen-e2555882-s003.pdf]

Data Sharing Statement

El-Bassel. Prevalence of Illicit Drug Detection in 5 US Cities Among Out-of-Treatment People Who Inject Drugs. *JAMA Netw Open*. Published February 05, 2026.
doi:10.1001/jamanetworkopen.2025.55882

Data
Data available: Yes
Data types: Deidentified participant data
How to access data: Data collected for this study may be made available on request. The data archive will be held at the Fred Hutch Cancer Center (Seattle, WA, USA). Requests can be sent to HPTN-Data-Access@scharp.org.
When available: beginning date: 12-31-2025

Supporting Documents
Document types: None

Additional Information
Who can access the data: Researchers whose proposed use of the data has been approved
Types of analyses: For a specified purpose
Mechanisms of data availability: After approval of a proposal, or with a signed data access agreement
